# Retraction Note: Nicotine promotes breast cancer metastasis by stimulating N2 neutrophils and generating pre-metastatic niche in lung

**DOI:** 10.1038/s41467-025-59975-w

**Published:** 2025-05-22

**Authors:** Abhishek Tyagi, Sambad Sharma, Kerui Wu, Shih-Ying Wu, Fei Xing, Yin Liu, Dan Zhao, Ravindra Pramod Deshpande, Ralph B. D’Agostino, Kounosuke Watabe

**Affiliations:** 1 Department of Cancer Biology, Wake Forest Baptist Medical Center, Winston-Salem, NC USA; 2 Biostatistics Core, Wake Forest Baptist Medical Center, Winston-Salem, NC USA

Retraction to: *Nature Communications* 10.1038/s41467-020-20733-9, published online 20 January 2021

The authors have retracted this article. After publication, concerns were raised regarding highly similar images in the figures, specifically:

- Fig. 1B ex vivo lung PBS Day 28 right image appears highly similar to Fig. 1H Day 10 (Pre-MET) +Nic. left image;
- Fig. 1H ex vivo lung Day 28 (MET) PBS left image appears highly similar to Supplementary Fig. S13C LCN2-KO left image;
- Four mouse images and one ex vivo lung image in Fig. 2A -Nicotine group appear highly similar to those in Supplementary Fig. S3A, representing different treatments;
- Fig. 3D Nic.+STATTIC middle mouse image appears highly similar to Fig. 6F Nicotine middle mouse image, with different fluorescence intensity;
- Fig. 4A MCF10CA1a Nic. CM image appears to overlap with Supplementary Fig. S10A MCF10CA1a Nic. CM image, representing cells treated with different conditioned media.
- Supplementary Fig. S1B Day 28 PBS right mouse image and Nicotine left mouse image appear highly similar, with different fluorescence intensity.

The authors have stated that these duplications occured due to errors in data handling, which may affect the reported results.

All authors agree to this retraction.

